# Phylogeography of the Indo-West Pacific maskrays (Dasyatidae, *Neotrygon*): a complex example of chondrichthyan radiation in the Cenozoic

**DOI:** 10.1002/ece3.448

**Published:** 2012-12-20

**Authors:** Melody Puckridge, Peter R Last, William T White, Nikos Andreakis

**Affiliations:** 1 Institute for Marine and Antarctic Studies, University of TasmaniaPrivate Bag 129, Hobart, TAS, 7001, Australia; 2 Wealth from Oceans Flagship, CSIRO Marine and Atmospheric ResearchCastray Esplanade, Hobart, TAS, 7000, Australia; 3 Australian Institute of Marine SciencePMB No. 3, Townsville, QLD, 4810, Australia

**Keywords:** Biodiversity hotspot, cryptic species, marine speciation, maskray, *Neotrygon*, phylogeography

## Abstract

Maskrays of the genus *Neotrygon* (Dasyatidae) have dispersed widely in the Indo-West Pacific being represented largely by an assemblage of narrow-ranging coastal endemics. Phylogenetic reconstruction methods reproduced nearly identical and statistically robust topologies supporting the monophyly of the genus *Neotrygon* within the family Dasyatidae, the genus *Taeniura* being consistently basal to *Neotrygon*, and *Dasyatis* being polyphyletic to the genera *Taeniurops* and *Pteroplatytrygon*. The *Neotrygon kuhlii* complex, once considered to be an assemblage of color variants of the same biological species, is the most derived and widely dispersed subgroup of the genus. Mitochondrial (COI, 16S) and nuclear (RAG1) phylogenies used in synergy with molecular dating identified paleoclimatic fluctuations responsible for periods of vicariance and dispersal promoting population fragmentation and speciation in *Neotrygon*. Signatures of population differentiation exist in *N. ningalooensis* and *N. annotata*, yet a large-scale geological event, such as the collision between the Australian and Eurasian Plates, coupled with subsequent sea-level falls, appears to have separated a once homogeneous population of the ancestral form of *N. kuhlii* into southern Indian Ocean and northern Pacific taxa some 4–16 million years ago. Repeated climatic oscillations, and the subsequent establishment of land and shallow sea connections within and between Australia and parts of the Indo-Malay Archipelago, have both promoted speciation and established zones of secondary contact within the Indian and Pacific Ocean basins.

## Introduction

Identifying and understanding biodiversity hotspots, regional endemicity, and their corresponding evolutionary drivers represent key elements to the long-term conservation of marine biota. The Indo-Australian Archipelago (IAA) is a geologically dynamic area of mega-diversity with species richness increasing in both latitudinal and longitudinal gradients toward the center of the region (Briggs 2000; Briggs 2005; Crame 2001). Peaks of species diversification have been reported within a number of marine groups coinciding with the arrival of the Australian Plate to the area, suggesting tectonic suture zones across the region underpin the area's biotic complexity (Santini and Winterbottom 2002; Renema et al. 2008; Williams and Duda 2008). Tectonic rafting, the establishment of new habitats in the form of shallow seas, tropical coastlines, island arcs, and the consequent changes in connectivity among islands coupled with glacio-eustatic sea level oscillations, has likely favored rapid diversification in a number of marine taxa by promoting vicariance and allopatric speciation (Crame 2001; Santini and Winterbottom 2002; Williams and Duda 2008; Carpenter et al. 2011).

Molecular phylogeography provides powerful insights into resolving complex genealogical relationships within so-called widely distributed taxa (Caputi et al. 2007; Fitzpatrick et al. 2011). In the IAA, phylogeographic studies have often revealed multiple cryptic species with restricted geographic distributions, strongly supporting the notion that this region acts as a cradle of species diversification. Demographic changes observed in marine taxa are often consistent with population contractions and expansions in response to sea-level oscillations during glacial cycles of the Pleistocene, for example, anemone and butterfly fishes (McMillan and Palumbi 1995), mantis shrimp (Barber et al. 2002, 2006), sea stars (Crandall et al. 2008), parrotfishes (Fitzpatrick et al. 2011). However, instances of inter- and intra-specific divergence recovered in other studies predate the Pliocene, indicating that historic and evolutionary processes responsible for systematic population fragmentation and the formation of this diversity center were catalytic on longer timescales, for example, fishes (Read et al. 2006), gastropods (Frey and Vermeij 2008), and squat lobsters, (Poore and Andreakis 2011). Among the growing body of literature exploring evolutionary patterns of species associated with this region, studies that investigate elasmobranch phylogeography are extremely rare, with the few examples either focused primarily on peripheral areas (e.g., Corrigan and Beheregaray 2009) or based on limited taxa with narrow coverage (e.g., Dudgeon et al. 2009).

The genus *Neotrygon* (maskrays) was previously considered a subgenus of *Dasyatis* before its recent elevation to generic level (Last and White 2008; Last et al. 2010). Maskrays are distinguished by a dark mask-like band over the eyes and represent an ideal group for understanding how evolutionary forces, geography, and environmental dynamics have shaped genetic structure, diversification, and population migration pathways in the IAA. First, they are confined to the Indo-West Pacific and four of the five nominal species exist as narrow-ranging endemics along the collision interface between the Australian and Eurasian tectonic plates (Last and Stevens 2009; Last et al. 2010). Second, they inhabit shallow coastal and continental shelf waters in <200 m depths (mostly <90 m) (Last and Stevens 2009). This makes them particularly sensitive to population fragmentation and lineage divergence due to environmental fluctuations, such as glacial advances and retreats or formation of phylogeographic breaks and contact zones.

Given that subtle body coloration differences between some species provide diagnostic characters useful for taxonomic purposes (Last and Stevens 2009), distinct color-morphs found in some *Neotrygon* species (White and Dharmadi 2007) may be indicative of cryptic species or represent historically isolated, geographically restricted, and reciprocally monophyletic populations of a single species that is Evolutionary Significant Units (ESUs, *sensu* Moritz (1994). Most notable is the blue-spotted maskray, *Neotrygon kuhlii* (Müller & Henle). Under existing taxonomic classifications, this species is considered common across the Indo-West Pacific region, representing an important component of artisanal and commercial fisheries in a number of areas (Compagno 1999; White and Dharmadi 2007). Although *N. kuhlii* is currently considered to be much more wide-ranging than the other four nominal species, distinct morphological and genetic variants occur throughout its range (White and Dharmadi 2007; Ward et al. 2008). These may correspond to cryptic species originated following recent fragmentation of a once pan-Indo-West Pacific population.

In this study, we use mitochondrial and nuclear molecular markers to explore how genetic diversity recovered within *Neotrygon* is geographically partitioned across the Indo-West Pacific (IWP). We aim to: (1) reconstruct genealogical relationships in *Neotrygon* at generic and species levels; (2) assess the taxonomic validity of morphs found within cosmopolitan morpho-species complexes and identify both cryptic speciation events and the geographic barriers that might have caused them. Thereafter, we apply a molecular clock approach to approximate the timing of divergence in *Neotrygon*. The resulting spatial and temporal patterns are interpreted in the light of the paleoclimatic and geological events driving vicariance and/or dispersal responsible for observed genetic discontinuities and the evolution of new maskray species.

## Materials and Methods

### Specimen collection and geographic locations

**Figure 1:**
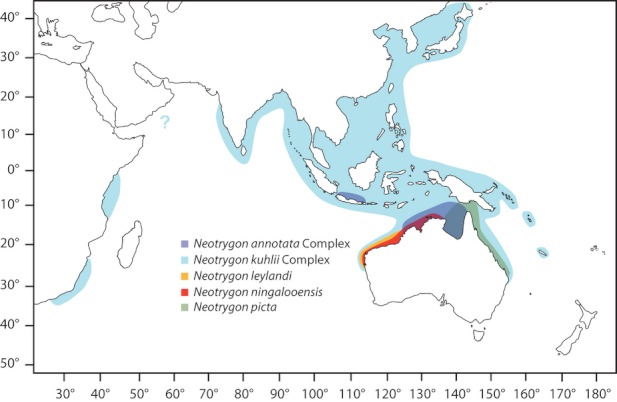
Known geographic distribution of *Neotrygon* species analyzed in this study adapted from Last and Stevens (2009) and Last et al. (2010).

Tissue samples from all nominal *Neotrygon* species across various locations throughout the IWP were assembled from the Australian National Fish Collection (ANFC) and contributions from colleagues. Whole specimen vouchers generally accompany samples (see Fig. 1 for known species distributions and Table S1 for sampling locations, geographic coordinates, and voucher information). Tissue samples were either preserved in 80% ethanol, 20% DMSO solution saturated with NaCl or frozen at −20°C prior to DNA extraction.

### DNA extraction, PCR amplification, and sequencing

Total genomic DNA was isolated from approximately 150 mg tissue following either Aljanabi and Martinez (1997) or Doyle and Doyle (1987) protocols. The partial mitochondrial cytochrome oxidase subunit I (COI) and ribosomal 16S rDNA gene fragments were PCR amplified using primers described in Ward et al. (2005) and Palumbi (1996), respectively. A fragment of the nuclear single locus recombination activating gene 1 (RAG1) spanning the more highly variable 5'-end of the gene was PCR amplified using primers Rag1For61 (Naylor pers. comm.) and Rag1Rev12 (Naylor et al. 2005), respectively (see Supplementary Information for PCR amplification conditions).

### Sequence alignments, data exploration, model, and outgroup selection

Electropherograms were edited in BioEdit v7.0.5 (Hall 1999); additional sequences were recovered from GenBank (http://www.ncbi.nlm.nih.gov) and the Barcode of Life databases (http://www.boldsystems.org) and aligned in MUSCLE (http://www.ebi.ac.uk/Tools/msa/muscle). Alignments were refined by eye.

Phylogenetic signal versus noise was assessed by computing *g*1 values from 10,000 random parsimony trees in PAUP* 4.0b10 (Swofford 2003), a measure of tree-length distributions (Hillis and Huelsenbeck 1992) and by estimating substitution saturation at codon positions using K2P distances (Kimura 1980) in DAMBE (Xia and Xie 2001). Diversity and alignment statistics were calculated in DnaSP v5.10.01 (Librado and Rozas 2009). Hierarchical Likelihood Ratio Tests (hLRTs) were computed in Modeltest Version 3.7 (Posada and Crandall 1998) to identify the best-fitting model and parameters given the alignment. To accept a concatenated phylogenetic analysis, single-marker Bayesian phylogenies were assessed for congruence based on topology and node support.

Phylogenies were inferred from: (1) the concatenated mitochondrial COI/16S; (2) the nuclear RAG1; and (3) the concatenated COI/16S/RAG1 alignments (referred to as mitochondrial, nuclear, and concatenated alignments, respectively). Outgroup comparisons were made against Dasyatidae genera in addition to the white-spotted guitarfish *Rhynchobatus australiae* (Whitley) (Rhynchobatidae), considered to be a stingray ancestral relative (Shirai 1996). The bull shark *Carcharhinus leucas* (Müller & Henle) was used for RAG1 phylogenies as *R. australiae* RAG1 sequences did not amplify successfully. To increase resolution of relationships, a separate phylogenetic analysis on the *N. kuhlii* complex was run using *N. leylandi* (Last) and *N. picta* Last & White as outgroups.

### Phylogenetic analyses and genealogical network reconstruction

We initially screened the mitochondrial phylogenies for historically isolated, geographically restricted, and reciprocally monophyletic clusters of sequences characterized by small intra-clade divergence and strong nodal support. Given these considerations, some of the identified clades could equate to Evolutionary Significant Units (ESUs, *sensu* Moritz 1994) deserving of independent conservation status. Mitochondrial DNA is commonly used in reconstructing genealogical relationships at the intra-specific level or among closely related taxa due to its fast evolutionary rate and recent coalescence time (Avise 1994). However, it is expected that gene genealogies inferred from taxa undergoing rapid speciation will vary significantly between loci, thereby compromising the accuracy of coalescent-based analyses and creating topological artifacts (Karl et al. 2012). Therefore, a comparison of multiple gene regions is necessary to resolve the demographic history of recently fragmented populations or newly emerging species (Edwards and Beerli 2000). Given these considerations, together with the limited number of nuclear markers used in this study, we use the term ‘clades’ instead of ‘ESUs’ to refer to the array of mitochondrial haplotype assemblages observed within *Neotrygon* species.

Maximum Likelihood (ML) and Bayesian Inferences (BI) were fixed with models and parameters identified by Modeltest and computed in PAUP* and MrBayes v3.1 (Ronquist and Huelsenbeck 2003), respectively. For ML, heuristic searches were run using the TBR branch-swapping algorithm with random addition of sequences. The GTR + Γ+ I model was used for concatenated analyses as PAUP* does not allow partitioning by loci. Bootstrap values were obtained from 10,000 random trees under the same ML model constraints used to infer tree topologies coupled with a distance based, neighbor-joining search option. For posterior probability estimates of nodes, priors for each locus were set individually using the output from Modeltest. We used the GTR model when the Modeltest output could not be performed in MrBayes, treating model parameters as unknown variables with uniform default priors. The Markov Chain Monte Carlo (MCMC) algorithm was used to sample across two parallel runs of four chains each running for 10 million generations sampling every 1000th. Standard deviations of split level frequencies were all <0.005 indicating convergence. The initial 25% of each sample run was discarded as burn-in.

Genealogical networks were constructed for *N. annotata* (Last), *N. kuhlii,* and *N. ningalooensis* Last, White & Puckridge using the median-joining algorithm (ε = 0, equally weighted characters) in Network v4.5.1.6 (http://www.fluxus-technology.com). The method groups related haplotypes through median vectors into a tree or network. Ambiguous relationships were resolved with a Maximum Parsimony (MP) heuristic algorithm. Median vectors can be interpreted biologically as extinct individuals or un-sampled haplotypes (Bandelt et al. 1999).

### Partition of genetic diversity & barriers to gene-flow

Levels of genetic divergence among genetically distinct lineages were first explored in MEGA v5.0 using the K2P-correction and Mantel tests were then run in Mantel v2.0 (Liedloff 1999) to test for isolation by distance for *N. kuhlii*. Furthermore, genetically distinct clades were split into their geographic sampling locations, such that clade 1, for example, comprised groups from Malaysian Borneo, Taiwan, Gulf of Thailand, and Vietnam. Pairwise genetic distances among these clades were then compared against Euclidean distances using 10,000 random iterations. Finally, barriers to gene-flow were identified in Barrier v2.2 (Manni et al. 2004). Geographic coordinates of sample locations were used to construct Delaunay triangulations (connecting sample locations) and Voronoï tessellations (intersecting the triangulations). The TN93 (Tamura and Nei 1993) genetic distance for all neighboring populations was calculated and linked to each edge of the Delaunay network (Manni et al. 2004). Barriers were then identified with Monmonier's maximum-difference algorithm along the edges of the network. The number of average pairwise genetic differences between clades above the overall mean was used to define *a priori* the number of genetic barriers.

### Past demographic history

Past demographic patterns were explored only for the genetically distinct clades with >5 specimens to assess: (1) neutrality of sequence evolution by means of Fu's *F*_S_ (Fu 1997) and the *R*_2_ statistic (Ramos-Onsins and Rozas 2002) in DnaSP v5.10.01; and (2) clade expansion by means of mismatch distributions (Harpending 1994) under a demographic expansion model using 1000 bootstrap replicates to calculate confidence intervals. To estimate past population dynamics through time from sequences of the *N kuhlii* complex taken as a whole, Extended Bayesian Skyline Plots (EBSP; the Bayesian coalescent inference of population dynamics) were computed in BEAST v1.6.1 (Drummond and Rambaut 2007). The EBSP accommodates multiple loci in a single analysis, thus reducing the coalescent error, and is suitable for taxa with complex histories and/or shallow phylogenetic structure. MCMC simulations were run for 25 million generations, sampling every 1000th for the COI, 16s, and RAG1 alignments using calculated mutation rates (described below). A strict molecular clock was enforced. Convergence and credibility intervals of the EBSP run were visualized in Tracer v1.4 (Rambaut and Drummond 2007).

### Divergence time estimates

In molecular phylogeography, divergence time approximations from intra-specific data and multiple, unlinked, concatenated loci are problematic because gene divergence almost always predates the divergence of populations. Furthermore, gene genealogies vary stochastically among unlinked loci and each locus is characterized by a different coalescent time independently of the historic events that have shaped the full genome (Edwards and Beerli 2000; Edwards et al. 2007). Phylogenetic reconstructions will therefore almost always reflect the gene genealogies rather than the species phylogeny.

We have calibrated our molecular clock against fossil data and published substitution rates and we have used BEAST v.1.6.1 to compute divergence time estimates by concatenating mitochondrial and nuclear markers in a single alignment. We acknowledge the aforementioned risks associated with dating nodes on gene genealogies rather than species phylogenies. However, our data included multiple species rather than multiple populations of the same species, a situation in which standard coalescent models are not appropriate. We have further increased accuracy of the computation by allowing each of the loci to evolve according to separate models and parameters.

Dasyatidae fossil records are extensive and well documented with the earliest known fossil dating to the Hauterivian age (130–136.4 Ma; early Cretaceous, Underwood et al. 1999). This and many early fossils are from teeth (Marmi et al. 2010) with paleontologists often assigning them to *Dasyatis* for convenience rather than from established taxonomic characters (Underwood et al. 1999). Given this, and the taxonomic uncertainties in extant fauna (e.g., Lovejoy 1996), the earliest record was used to set a minimum age for Dasyatidae using the concatenated dataset. Additional divergence estimates were inferred from the mitochondrial alignment using mitochondrial mutation rates (0.58% My and 0.90% My) of the spotted eagle ray *Aetobatus narinari* (Euphrasen) approximated on the basis of the final Isthmus of Panama closure (Richards et al. 2009). Deviations from clock-like substitution rates were assessed in PAUP* using the likelihood ratio test (LRT; Felsenstein 1981). Molecular clock computations were performed in BEAST v1.6.1 under a relaxed clock assumption to allow branches to vary among lineages according to an uncorrelated lognormal distribution (Drummond et al. 2006). The tree prior was defined using the Yule Process. MCMC simulations were run for 25 million iterations and convergence was checked in Tracer v 1.5.

## Results

### Phylogenetic reconstructions

**Figure 2:**
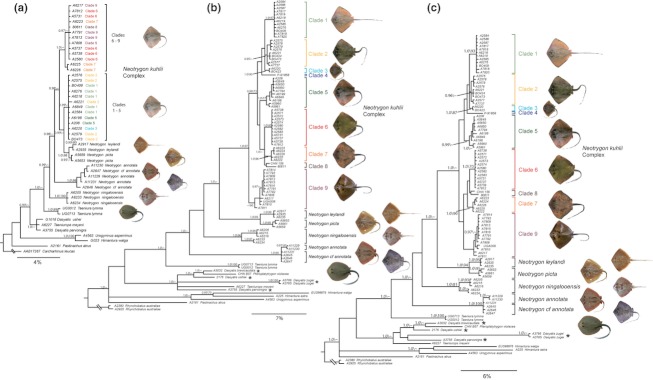
Bayesian phylogenetic hypothesis of *Neotrygon* species and genera of Dasyatidae inferred from: (a) the partial RAG1 gene; (b) the concatenated mitochondrial COI and 16s genes; and (c) the concatenated COI, 16s, and RAG1 genes. Node support of ≥0.95 posterior probability and ≥70% bootstrap support are given, respectively, with ∼ denoting support below these threshold values. Stars denote polyphyletic relationships within *Dasyatis*.

A total of 95, 92, and 47 sequences were collated for the COI, 16s, and RAG1 alignments, respectively (see Table S1 for sequence accession numbers; Table 1 for alignment statistics and model selection). Distribution of 10,000 random trees was considerably left-skewed for all alignments indicating strong phylogenetic content; substitution saturation was evident only in the third codon position of the COI gene at the family level (Fig. S1–S3). This was left to evolve independently in BI to reduce its influence on the topology. All markers produced nearly identical and robust topologies (Fig. 2a–c) supporting the monophyly of *Neotrygon* within the family Dasyatidae, the genus *Taeniura* as basal to *Neotrygon,* and *Dasyatis* as polyphyletic to the genus *Pteroplatytrygon* in mitochondrial and concatenated analyses (*Pteroplatytrygon* did not amplify in RAG1 for nuclear comparison). Although *Neotrygon* genealogies were largely concordant among datasets, the position of either *Neotrygon annotata* or *N. ningalooensis* as ancestral forms to the *Neotrygon* group (Fig. 2a–c) is uncertain.

**Table 1:** Alignment statistics for COI, 16s, RAG1, and combined datasets calculated from the global dataset (Dasyatidae) and only for the *Neotrygon kuhlii* species complex

Alignment	*n*	Length (bp)	Model	Invariant Sites (I)	Gamma shape (α)	π	*h*	PI	*g*1
Dasyatidae									
COI	95	651	TrN + I + Γ	0.5669	1.1819	0.08963	(48) 0.963	242	−0.579033
16s	92	596	GTR + I + Γ	0.4903	0.5194	0.03998	(29) 0.936	21	−0.704759
RAG1	47	953	HKY + Γ		0.6662			19	−1.140617
Mitochondrial	98	1247	GTR + I + Γ	0.5839	1.0121				−0.546615
Combined	98	2200	GTR + I + Γ	0.5869	0.6451				−0.604791
*N. kuhlii*									
COI	65	651	HKY + Γ		0.1578	0.03177	(23) 0.919	66	−0.577075
16s	64	596	TrN + I	0.8322		0.01218	(15) 0.885	28	−0.549506
RAG1	29	953	HKY + I	0.9609				13	−0.480178
Mitochondrial	68	1247	GTR + I + Γ	0.6588	0.8443				−0.521827
Combined	68	2200	GTR + I + Γ	0.7924	0.6263				−0.507290

*n*, number of sequences; π, nucleotide; and *h*, haplotype diversities; PI, proportion of parsimony informative sites; *g*1, distribution skewness of 10,000 random trees.

### Species level phylogenies and clade delineations in *Neotrygon*

Molecular phylogenies fully supported morphological characterization of currently recognized species (Fig. 2a–c). However, remarkable genetic discontinuities were encountered within the morphologically distinct taxa *N. kuhlii* (1.33% to 4.62%)*, N. ningalooensis* (4.75%), and, to a lesser extent, *N. annotata* (0.46%) (Tables S2, S3). The recovered clusters of sequences found across these species corresponded to genetically distinct, reciprocally monophyletic, historically isolated lineages. Furthermore, they showed phylogeographic concordance in all cases except for the clades of *N. annotata* in the Gulf of Carpentaria and the clades of *N. kuhlii* occupying the Sunda Shelf. The sympatric distributions of the aforementioned clades in these two regions are likely the result of secondary contact.

**Figure 3:**
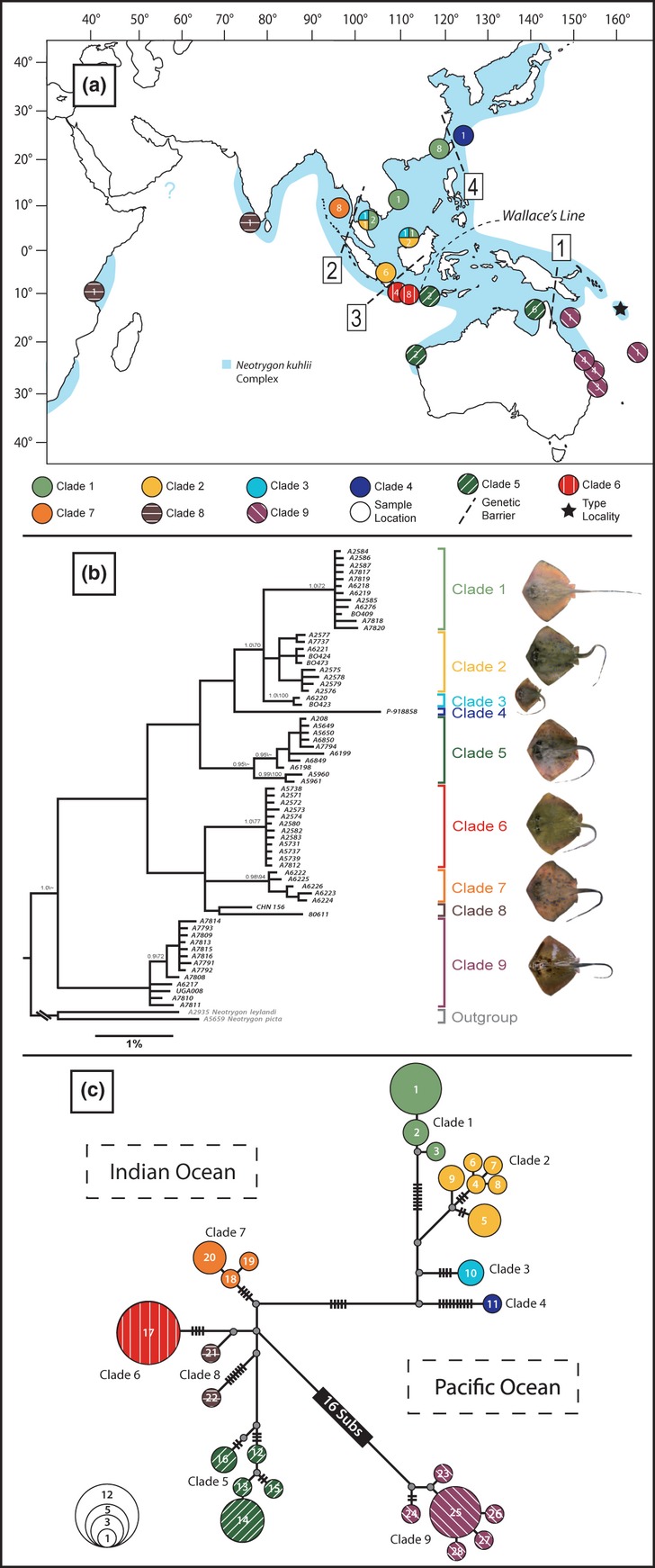
Genealogical relationships within the *Neotrygon kuhlii* complex. In (a) geographic distribution of *N. kuhlii* clades 1–9 with numbers of samples given at each location; dashed lines denote geographic barriers responsible for genetic discontinuities in descending order from 1 to 4. In (b) Bayesian phylogenetic hypothesis of clades based on the concatenated mitochondrial and nuclear dataset; numbers on nodes indicate posterior probabilities and bootstrap support, respectively. In (c) COI median-joining networks illustrating genealogical relationships among haplotypes of clades 1 to 9. Circles indicate haplotypes; lines denote substitution steps among haplotypes; the size of the circles is proportional to the frequency of the haplotypes; gray circles represent median vectors of either un-sampled or extinct taxa.

Nine reciprocally monophyletic mitochondrial clades, regrouped into two main lineages, were observed within *N. kuhlii* (1–9; Fig. 3a–c). Clades 1–5 occur across the northwest Pacific and northwestern Australia; clades 6–9 are found in the Indian Ocean and eastern Australia (Fig. 3a). The nuclear RAG1 gene strongly supported the two major clusters, yet this marker was unable to resolve individual clades. Placement of clades 5 and 9 shifted in the mitochondrial datasets (Fig. 2b). Clade 5 clustered with clades 6–8 across the Indian Ocean rather than clades 1–4 (Fig. 2c); however, this relationship lacks strong internal node support. Clade 9 is highly supported as ancestral to the entire *N. kuhlii* complex (Fig. 2b) in the concatenated mitochondrial phylogeny; clades 1–3 co-occur sympatrically while all other *N. kuhlii* clades show allopatric distributions.

**Figure 4:**
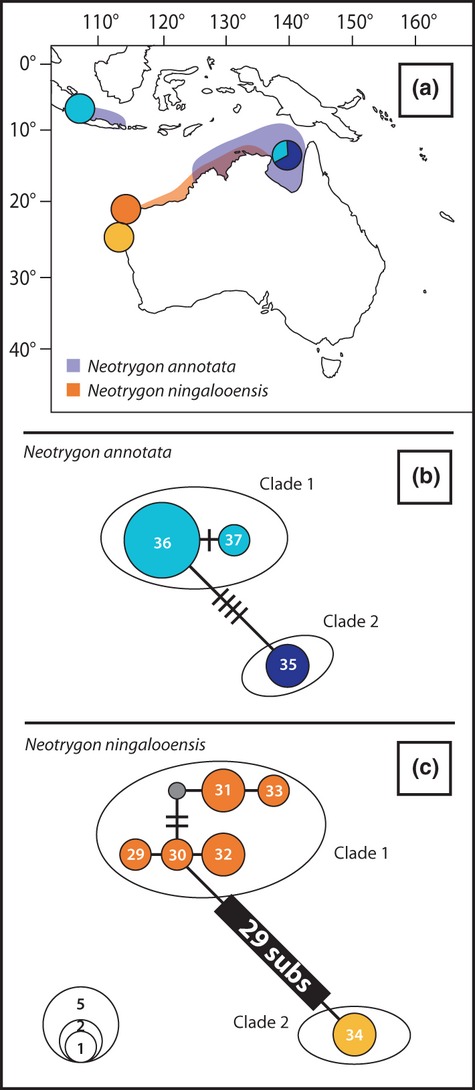
*Neotrygon ningalooensis* and *N. annotata* complexes. In (a) their known geographic distributions and haplotype sampling locations; in (b) and (c) the genealogical relationships among haplotypes. Circles indicate haplotypes; lines denote substitution steps among haplotypes; the size of the circles is proportional to the frequency of the haplotypes; gray circles represent median vectors of either un-sampled or extinct taxa.

Two mitochondrial clades are found within *N. ningalooensis* in Western Australia: one in Coral Bay, the other in Shark Bay ca. 300 km apart (Fig. 4a, c). These were not supported by the nuclear marker (Fig. 2a). In contrast, two mitochondrial clades recovered in *N. annotata* (Fig. 4a, b) were supported by the nuclear RAG1 gene (Fig. 2a). *Neotrygon picta* and *N. leylandi*, endemic to the northeastern and northwestern coasts of Australia, respectively, were recovered sister and basal to *N. kuhlii* (Fig. 2b, c), yet paraphyletic to the latter in the nuclear RAG1 phylogenies (Fig. 2a).

Overall, the mitochondrial data produced in this study seems to satisfy conditions for identification of ESUs, characterized by historic isolation and therefore distinct evolutionary potential in the vast majority of cases (Moritz 1994). However, despite the high-resolution power of the mitochondrial markers, the clusters recovered in this study received limited support from our sole nuclear marker (i.e., the nine *N. kuhlii* mitochondrial clades collapse into two nuclear clusters of sequences, likely representing two biologically distinct species). We therefore acknowledge that the observed gene genealogies may simply represent multiple mutations rather than ESUs within the evolutionary trajectory of these taxa.

### Network analysis

Median-joining network analysis of 601 bp of the trimmed COI alignment revealed 10 frequent haplotypes (>1 individuals) and 18 singletons in *N. kuhlii* (Fig. 3c). Haplotypes of clade 8 were highly divergent (Hap21, India *vs*. Hap22, Tanzania, ca. 4,500 km apart); clades 2, 5, and 9 were the most variable; clades 3, 4, and 6 consisted of a single haplotype (Fig. 3c; Table 2). The two *N. ningalooensis* haplogroups were separated by 29 mutational steps (Fig. 4c); less divergence separated *N. annotata* haplogroups between the Gulf of Carpentaria and eastern Indonesia (Fig. 4b). Interestingly, both *N. annotata* mitochondrial haplogroups co-occur in the Gulf of Carpentaria (Fig. 4a).

**Table 2:** Alignment statistics of the trimmed COI gene fragment of *Neotrygon kuhlii* clades

Clade	*n*	*h*	*π*	*F*_S_	*R*_2_
All	63	(28) 0.934		−0.353	0.09962
1	10	(3) 0.511	0.00088	−0.594	0.1744
2	9	(6) 0.889	0.00572	−0.662	0.1777
3	2*	N/A	N/A	N/A	N/A
4	1*	N/A	N/A	N/A	N/A
5	10	(5) 0.756	0.00503	0.897	0.1598
6	12	(1) 0.000	0.00000	–	–
7	5	(3) 0.700	0.00133	0.276	0.2833
8	2*	N/A	N/A	N/A	N/A
9	12	(6) 0.682	0.00303	−1.433	0.1275

*n*, number of sequences; *h*, number of haplotypes (in brackets) and haplotypic diversity; π, nucleotide diversity; Fu's *F*_S_; Ramos-Onsins and Roza's *R*_2_ statistics.

* Sample numbers <5 and therefore insufficient for analyses.

### Barriers to gene-flow within *N. kuhlii*

Average K2P-corrected distances ranged from 0.00% (within clades 3 and 6) to 1.56% (within clade 8) and from 1.33% (between clades 2 and 3) to 4.62% (between clades 1 and 9) for the *N. kuhlii* complex (Table S2). The minimum inter-specific *Neotrygon* divergence was 2.73%, between *N. leylandi* and *N. picta* (Table S3). Genetic distances among clades correlated well with their geographic distribution, according to the Mantel tests (*r* = 0.5486, *P* < 0.005). On the other hand, the sympatric distribution of some clades (Fig. 3a) suggested that isolation by distance is not the sole factor responsible for the partition of genetic variation.

The most prominent barriers responsible for interrupting gene-flow within the *N. kuhlii* species complex are localized in the IAA (Fig. 3a). Torres Straight represents the first biogeographic barrier separating clade 9 from all other clades. A second barrier separates clades 7 and 8 from the Andaman coast of Thailand westward across the Indian Ocean. A third barrier intersects the Indonesian Archipelago between clade 6 in Central Java from clade 2 collected from the Muara Angke fish market in West Java, which are supplied by fishing grounds off southeast Sumatra. Finally, a fourth geographic barrier, located across Ishigaki Island in the East China Sea, separates clade 4 from the rest of the clades.

### Past population demographic history and divergence time estimates

**Figure 5:**
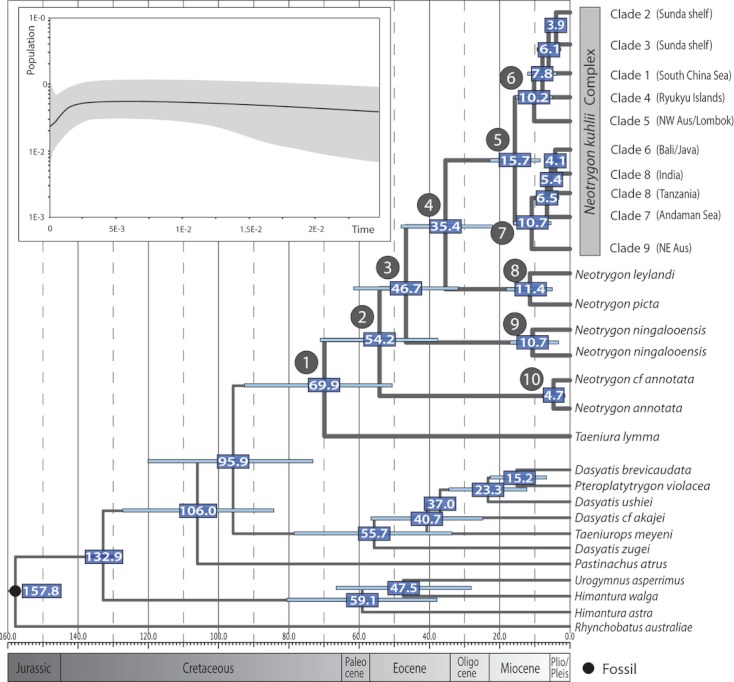
Divergence time approximations within Dasyatidae calibrated against fossil records with 95% credibility intervals in major nodes (numbered 1 to 10). The black dot denotes the calibration fossil. A timescale in My before present and a geological timescale is given below. Ages of nodes 1 to 10 are detailed in Table S4 for comparison with dates based on COI mutation rates. A Bayesian skyline plot derived from *Neotrygon kuhlii* COI sequences is depicted on the left. Black line denotes fluctuations of the median effective population size; the shaded area represents 95% confidence range. Time in thousands of years (Ky) is reported in the *x*-axis.

Whether considering *N. kuhlii* as a panmictic group or as nine separate mitochondrial lineages, neutrality tests of Fu's *F*_S_ and the *R*_2_ statistic showed non-significant departures from expectations (Table 2). Mismatch distributions also revealed no population changes for these clades (Fig. S4); yet, the EBSP analysis highlighted a consistent population size through time followed by a dramatic decline over the past ∼350,000 years (Fig. 5).

**Figure 6:**
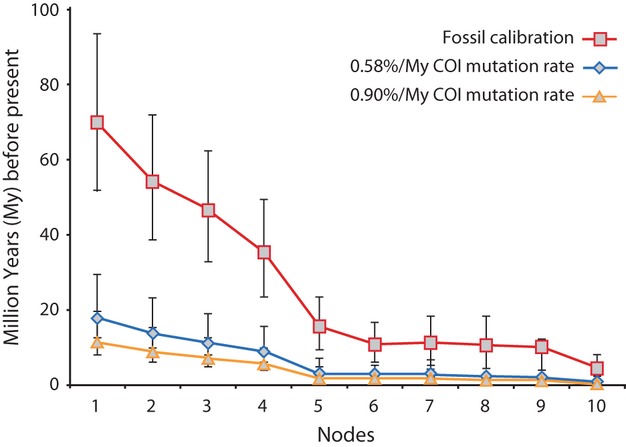
Mean divergence times of nodes 1 to 10 based on the fossil calibration of this study and mitochondrial mutation rates of the spotted eagle ray *Aetobatus narinari,* (Richards et al. 2009) set at 0.58% per My and 0.90% per My on the basis of conflicting estimates of the final closure of the Isthmus of Panama.

Fossil-calibrated divergence time approximations yielded mutation rates of 0.229%, 0.078%, and 0.022% per My for the COI, 16s, and RAG1 markers, respectively. *Neotrygon* diverged from *Taeniura* in the early Eocene (70–40 Ma). A series of rapid cladogenesis events over the last 15 My were responsible for: (1) the separation between Indian and Pacific *N. kuhlii* clades; (2) the lineage splits within *N. ningalooensis* and *N. annotata;* and (3) between the geminate species *N. picta* and *N. leylandi*. These divergence dates provide an averaged 4-fold increase in age estimates to those based on the mitochondrial mutation rates of *A. narinari*. These rates place the origin of *Neotrygon* in the early to mid-Miocene (17.9 or 11.6 Ma for the 0.58% and 0.9% per My rates, respectively) with the major cladogenesis events across the group occurring throughout the Plio-Pleistocene (Fig. 6; Table S4).

## Discussion

Mitochondrial and nuclear phylogenies, inferred from multiple individuals representing all nominal species of *Neotrygon*, were completely resolved, confirming their recent generic-level assignment (Last and White 2008) and monophyly. The narrow-ranging endemics along the Australian Plate were recovered ancestral to the widespread *N. kuhlii* group, suggestive of Austral origins (Fig. 2). As their center of origin and center of diversity appear to overlap, one possible explanation is that the *Neotrygon* rays migrated to the region via tectonic rafting, arriving with the Australian Plate. Subsequent development of extensive shallow sea habitat, forming dispersal pathways for ancestral *N. kuhlii* populations, may have therefore been instrumental in their success in colonizing coastal regions of the tropical and subtropical IWP. Sharp genetic discontinuities observed in *N. kuhlii*, *N. ningalooensis,* and *N. annotata* suggest that genetically homogeneous species, once occupying larger geographic ranges than at present, have been fragmented via rounds of major paleoclimatic and geological events. We briefly discuss the taxonomic implications of our findings and thereafter focus on the evolutionary mechanisms that are likely responsible for the formation of new maskray species in the IAA.

### Taxonomic considerations

The blue-spotted fantail ray *Taeniura lymma* (Forsskål) was recovered basal to *Neotrygon* rather than *Dasyatis*, the group to which the *Neotrygon* rays were previously assigned (e.g., Compagno and Heemstra 1984). *Dasyatis* has long been recognized on the basis of poorly defined characters (e.g., Lovejoy 1996). The polyphyletic status of the genus identified here supports Last and White's (2008) suggestion that a taxonomic revision for this group is needed (Fig. 2). The genus *Taeniurops*, once assigned to *Taeniura*, gained molecular support as a separate genus more closely related to *Dasyatis* than to *Taeniura* (Fig. 2).

A character state shared between *T. lymma* and some of the *Neotrygon* species, particularly with members of the *N. kuhlii* complex, is the presence of blue spots, or ocelli, decorating their dorsal surface. *Taeniura lymma* can be distinguished from *N. kuhlii sensu lato* by its oval-shaped disk and longer tail with a deep ventral skin fold reaching to the tip (Last and Stevens 2009). Blue-spotted disks are also found to a lesser extent in *N. ningalooensis* (Last et al. 2010), and a partial absence of blue spots is seen in *N. kuhlii* mitochondrial clade 1. Blue spots appear to be a polyphyletic character and probably evolved more than once in stingrays. Similar convergent color patterns have been reported in a variety of other vertebrate groups and are believed to be involved in processes such as intra-specific communication, response to the environment, or predator–prey interactions (Seehausen et al. 1999; Manceau et al. 2010; Rosenblum et al. 2010).

### Species diversity in *Neotrygon*

Divergent populations within fishes are increasingly regarded as separate species (Eschmeyer et al. 2010). Eighty new elasmobranch species have been discovered or named between 2005 and 2009 in the IAA, indicating a large proportion of species diversity in the region has been overlooked until recently (Last and Stevens 2009). Within *Neotrygon,* body coloration and COI barcoding have been instrumental in identifying cryptic species (Ward et al. 2008; Last et al. 2010). A divergence level of 2% in COI is sufficient to delineate the great majority of fish and elasmobranch taxa (Ward et al. 2005; Puckridge et al. 2013). However, elasmobranchs typically exhibit much lower values of genetic diversity at the within-species level. For example, following pairwise comparisons of COI divergences in 171 elasmobranch species, less than 1% within-species variation was observed in 93% of cases (Ward et al. 2008). Only 2.73% COI sequence divergence discriminates sister species *N. leylandi* and *N. picta*.

The levels of mitochondrial COI differentiation in *N. kuhlii*, *N. ningalooensis,* and, to a lesser extent, *N. annotata*, coupled with previous observations of morphological differences (see e.g., White and Dharmadi 2007), suggest multiple cryptic species. However, this same diversity is not reflected in the nuclear RAG1 gene. Instances of incongruence between mitochondrial and nuclear markers in some elasmobranchs have been attributed to sex-biased dispersal (Dibattista et al. 2008; Portnoy et al. 2010). This is because males can have wide-range breeding strategies resulting in genetically homogeneous populations, inferred from nuclear phylogenies, over larger areas. In contrast, females can show strong site fidelity to nursery grounds and therefore display high geographic structuring as inferred by mitochondrial markers. Site fidelity for long periods has been reported in both male and female *N. kuhlii* tagged on the east coast of Australia (Pierce et al. 2009) making this an unlikely scenario for this group. A more parsimonious explanation for this incongruence relates to the inherent differences between the mitochondrial and nuclear genomes themselves. Mitochondrial regions mutate at an order of magnitude faster than nuclear regions, thereby increasing their response to fragmentation. In addition, they have one-quarter the effective population size (*N*_e_) and therefore higher fixation potential of alleles due to genetic drift (Larmuseau et al. 2010). The nuclear RAG1 gene is considered an effective marker at reconstructing elasmobranch phylogenies at higher taxonomic levels (Naylor et al. 2005; López et al. 2006). Despite its slow evolutionary rate (Schluter and Marchalonis 2003), it resolved sister species *N. leylandi* and *N. picta*, characterized by similar COI divergences observed within *N. kuhlii* and *N. ningalooensis*. In the absence of detailed morphological knowledge, we opt for a conservative scoring of cryptic speciation events within *Neotrygon* based on the results from just the nuclear RAG1 gene and therefore infer two species of *N. kuhlii* (clades 1–5 and clades 6–9).

### Phylogeography and cryptic speciation of *Neotrygon* maskrays in the Pleistocene

Three morphotypes have been reported within the *N. kuhlii* complex: one off northern Australia (Last and Stevens 2009), another from Bali, but also observed off Lombok, and a third from Java (White and Dharmadi 2007; Ward et al. 2008). In this study, we confirm nine geographically structured mitochondrial lineages, distributed across the IWP corresponding to at least two distinct species (Fig. 3a–c). We interpret their spatial arrangement in the light of major biogeographic barriers and large-scale geological events.

#### The Torres strait

At current sea levels, this 5 to 25 m deep strait stretches 150 km and accounts for the complete isolation of clade 9 (the most pronounced genetic break within *N. kuhlii sensu lato*; Fig. 3a). A similar separation is evident in sister species *N. leylandi* and *N. picta* (fig. 1), although its location is displaced westward along the northwest coast. The strait acts as a periodic land barrier, having been exposed for the great majority of glacial cycles and sea level fluctuations over the past 250,000 years (Voris 2000). A complex system of seasonal currents and surface winds sustain a mostly westward flow, moving Pacific waters through this strait when it is flooded (Saint-Cast and Condie 2006). Varying genetic and geographic signatures of isolation across Torres Strait are apparent across diverse marine taxa. Factors such as differences in dispersal potential, habitat specificity, and level of exposure to the flow of water are invoked to explain these conflicting patterns (Mirams et al. 2011). Interestingly, isolation of *N. kuhlii* clade 9 from the other clades dates to a similar timeframe to that of *N. leylandi* from *N. picta* either in the mid-Miocene (∼10 Ma) or Plio-Pleistocene (∼3–1.5 Ma).

#### The Sunda arc

Similarly to the Torres Strait, sea level fluctuations have repeatedly exposed the Sunda Arc, along the Indian Ocean margin of the Sunda Shelf (Fig. 3a). Subsequent fragmentation between sea basin populations in marine species (Carpenter et al. 2011 and references therein) suggests that geographic barriers 2 and 3 (Fig. 3a) represent a single continuous vicariant break. A simultaneous interruption of gene-flow among historic *N. kuhlii* populations gains support from both mitochondrial and nuclear phylogenies, suggesting persistent periods of separation.

Discrete morphotypes of *N. kuhlii* have been observed along this arc (White et al. 2006). The two morphotypes occurring in Java and Bali correspond to clades 2 and 6, respectively. However, a third clade (clade 5, also distributed across northern/northwestern Australia) was found in Lombok rather than the Bali form as previously suggested (White and Dharmadi 2007). Either clades 5 and 6 share sympatric distributions, the fish markets in Tanjung Luar (Lombok) are intermittently supplied by catches from neighboring islands, or morphological differences between clades 5 and 6 are not readily apparent. As we have accurate information that this species is collected from small islands close to the Tanjung Luar landing site, translocation of the catch is an unlikely explanation.

#### The Lombok strait

The Lombok Strait separates clades 5 and 6. It is one of three main outflow passages of the Indonesian through-flow, transporting Pacific waters into the Indian Ocean (Sprintall et al. 2009) and also represents the southern end of the Wallace Line. This was traditionally considered a terrestrial biogeographic boundary; however, recent evidence suggests a marine equivalent exists (Lourie and Vincent 2004). The strong currents (mean flow rate of 2.6 million cubic meters/second, Sprintall et al. 2009) and deep waters of this strait pose a challenge for shallow water species. Genetic separation either side of this line was previously observed in the zebra shark *Stegostoma fasciatum* (Hermann) (Dudgeon et al. 2009), another demersal elasmobranch. Similar genetic discontinuities are expected between Indonesian and northern Australian populations as the Ombai Strait and Timor Passage, located further east, transport water at much higher rates than the Lombok Strait (Sprintall et al. 2009). Although genetic divergence between Lombok and northern Australia is evident, curiously, it does not reflect the same level of isolation separating Bali and Lombok and therefore does not support the Indonesian through-flow as a viable explanation for a marine Wallace Line. Marine studies that support an east to west separation across Wallace's Line may represent artifacts due to incomplete sampling across the region (Carpenter et al. 2011).

#### The Ryukyu Islands

The single specimen of clade 4 from Ishigaki Island (Ryukyu Island group) diverges 3.11% COI from the neighboring clade 1, distributed across the South China Sea and into Taiwan (Fig. 3a). The Okinawa Trench, separating Taiwan from the Ryukyu Islands, began its rifting about 2 Ma (Kimura 1985). This rifting, environmental fluctuations and the onset of the Kuroshio Current likely account for the isolation of clade 4. Divergence within clade 8 (between India and Tanzania) is not addressed due to lack of detailed sampling across its range. However, the large geographic distances separating these two regions may afford some explanation of their divergence.

### Rounds of population expansion–contraction and secondary contact in Pleistocene

The great majority of lineage diversification across IWP populations of *N. kuhlii* is fostered in the IAA. Patterns of fragmentation suggest that *N. kuhlii* populations were subject to historic rounds of expansion and contraction in line with periodic glacial maxima (Briggs 2000). Periods of contraction are characterized by population fragmentation followed by either extinction or sub-population survival in refugia zones. Following *de novo* range expansion during glacial minima, fusion of sub-populations will be favored in secondary contact zones. The sympatric distribution of clades 1, 2, and 3 across the northern Sunda shelf probably depicts recent secondary contact in response to flooding of the shelf since the last glacial period. These overlapping, yet genetically distinct lineages may either interbreed, forming reticulated histories, or be reproductively isolated in sympatry. Pre- or post-zygotic reproductive barriers are necessary to maintain their genetic integrity. Temporal variation in reproductive timing is not a likely scenario as asynchronous annual reproduction has been previously observed in adjacent *N. kuhlii* clades (White and Dharmadi 2007).

Observed color variation, that is, the partial absence of blue spots in clade 1 and variation between other clades (White et al. 2006), may offer potential for pre-zygotic isolation if it influences mate recognition. Little is known about the range of cues involved in mate recognition in elasmobranchs. A limited number of studies invoke pheromone-driven processes (Johnson and Nelson 1978; Kajiura et al. 2000), drawing on the highly developed olfactory senses typical of many elasmobranchs. However, unlike many other elasmobranch species, which cannot distinguish color, *N. kuhlii* have the appropriate hardware to see across the blue to red color spectrum (Theiss et al. 2007). Additionally, the convergence of color patterns observed across *Neotrygon* and their ancestors is a phenomenon that can arise through mate selection making it tempting to speculate that these color differences will foster further divergence in these sympatrically distributed clades.

### Phylogeography and population differentiation in *Neotrygon ningalooensis* & *N. annotata*

Populations of *N. ningalooensis* are highly restricted, found to only ∼5 m depths along the tropical west coast of Australia (Last et al. 2010). Steep bathymetric contours along this coastline represent a potential challenge for dispersal and connectivity among populations. Deep mitochondrial genetic divergence (4.75%) between *N. ningalooensis* clades (Fig. 4a; Table S3) suggests an interruption of gene-flow between populations during lowered sea levels in either mid-Miocene (∼10.7 Ma) or Plio-Pleistocene (3–2 Ma) (Fig. 5; Fig. 6). As the nuclear RAG1 gene does not differentiate these clades and no apparent morphological differences can distinguish them, their separation as biologically distinct species cannot be determined. Factors such as male-mediated gene-flow need to be investigated to explain this incredible discrepancy between mitochondrial and nuclear markers as well as use of additional, more informative nuclear markers that may help resolve some of the uncertainties around processes creating divergences in this group.

In contrast, although the amount of sequence variation in *N. annotata sensu lato* is small, separation is reflected in both mitochondrial and nuclear markers. The two clades evident also maintain genealogical concordance where they co-occur sympatrically in the Gulf of Carpentaria, lending weight to their recognition as separate species. Timing of their divergence is consistent with the climatically driven coastline contractions of the Plio-Pleistocene.

## Conclusions

Coastal marine habitats support greater levels of marine diversity than their pelagic or deep-sea counterparts and the associated diversity levels correlate well with sea-surface temperature and coastline length (Tittensor et al. 2010). At least three marine biodiversity hotspots have evolved within the last 50 My; their timing and location coincide with major tectonic events (Renema et al. 2008). Origins of the genus *Neotrygon* appear to be austral with the ancestral, narrow-ranging, endemics centered on the Australian Plate, highlighting the potential contribution of the Australian biota in fueling this mega-diverse region. Subsequent dramatic sea-level changes, as seen in the mid-Miocene and throughout the Plio-Pleistocene (Voris 2000), appear to have fragmented a once large and continuous *N. kuhlii* population. Genetic signatures across *Neotrygon* and present-day distributions are consistent with the survival of genetic variants of the species in isolated refugia, emphasizing the role of historic climatic events in shaping modern maskray diversity patterns within the IAA.

## Data accessibility

DNA sequences used in this study correspond to Genbank accession numbers found in supporting information Table S1.

## Supporting Information

**Table S1.** Specimens used in this study; accession numbers of sequences and collection information. Details of the COI haplotypes are also given for the specimens of *Neotrygon annotata, N. kuhlii*, and *N. ningalooensis*.

**Table S2.** Kimura 2-Parameter (K2P) corrected Neighbor-Joining distance matrix with minimum and maximum values (in brackets) for the *Neotrygon kuhlii* mitochondrial COI clades 1 to 9.

**Table S3.** K2P-corrected neighbor-joining distance matrix of *Neotrygon* species calculated from the COI alignment. Genetic distance is along the lower diagonal and standard error along the upper diagonal.

**Table S4.** Mean divergence times and their 95% HPD confidence intervals based on fossil calibrations and published COI mutation rates (0.58%/My and 0.9%/My) calculated in the spotted eagle ray (*Aetobatus narinari*) from the isthmus of panama closure for nodes 1 to 10.

**Figure S1.** Substitution frequencies of transitions (cross) and transversions (triangle) for: a) the 1st, b) 2nd, and c) 3rd codon positions of the partial COI gene. Observed frequencies are calculated with the K2P genetic distance.

**Figure S2.** Substitution frequencies of transitions (cross) and transversions (triangle) for: a) the 1st, b) 2nd, and c) 3rd codon positions of the partial 16s gene. Observed frequencies are calculated with the K2P genetic distance.

**Figure S3.** Substitution frequencies of transitions (cross) and transversions (triangle) for: a) the 1st, b) 2nd, and c) 3rd codon positions of the partial RAG1 gene. Observed frequencies are calculated with the K2P genetic distance.

**Figure S4.** Mismatch distributions for pairwise comparisons of *Neotrygon kuhlii* COI mitochondrial clades. Monomorphic clades 3, 4, and 6 were excluded from the calculation. The frequencies of expected and observed differences are given as a solid and dashed line, respectively.

## References

[b1] Aljanabi SM, Martinez I (1997). Universal and rapid salt-extraction of high quality genomic DNA for PCR-based techniques. Nucleic Acids Res.

[b2] Avise JC (1994). Molecular markers, natural history and evolution.

[b3] Bandelt HJ, Forster P, Röhl A (1999). Median-joining networks for inferring intraspecific phylogenies. Mol. Biol. Evol.

[b4] Barber PH, Palumbi SR, Erdmann MV, Moosa MK (2002). Sharp genetic breaks among populations of *Haptosquilla pulchella* (Stomatopoda) indicate limits to larval transport: patterns, causes, and consequences. Mol. Ecol.

[b5] Barber PH, Erdmann MV, Palumbi SR (2006). Comparative phylogeography of three codistributed stomatopods: origins and timing of regional diversification in the Coral Triangle. Evolution.

[b6] Briggs JC (2000). Centifugal speciation and centres of origin. J. Biogeogr.

[b501] Briggs JC (2005). The marine East Indies: diversity and speciation. J Biogeogr.

[b7] Caputi L, Andreakis N, Mastrototaro F, Cirino P, Vassillo M, Sordino P (2007). Cryptic speciation in a model invertebrate chordate. Proc. Natl Acad. Sci.

[b8] Carpenter KE, Barber PH, Crandall ED, Ablan-Lagman MCA, Ambariyanto, Mahardika GN (2011). Comparative phylogeography of the Coral Triangle and implications for marine management. J. Mar. Biol.

[b9] Compagno LJV, Carpenter KE, Niem VH (1999). General remarks: batoid fishes. FAO species identification guide for fishery purposes. The living marine resources of the Western Central Pacific.

[b10] Compagno LJV, Heemstra PC (1984). *Himantura draco*, a new species of stingray (Myliobatiformes: Dasyatidae) from South Africa, with a key to the Dasyatidae and the first record of *Dasyatis kuhlii* (Müller & Henle, 1841) from southern Africa. J.L.B. Smith Inst. Ichthyol. Spec. Publ.

[b11] Corrigan S, Beheregaray LB (2009). A recent shark radiation: molecular phylogeny, biogeography and speciation of wobbegong sharks (family: Orectolobidae). Mol. Phylogenet. Evol.

[b12] Crame JA (2001). Taxonomic diversity gradients through geological time. Divers. Distrib.

[b13] Crandall ED, Jones ME, Munoz MM, Akinronbi B, Erdmann MV, Barber PH (2008). Comparative phylogeography of two seastars and their ectosymbionts within the Coral Triangle. Mol. Ecol.

[b14] Dibattista JD, Feldheim KA, Thibert-Plante X, Gruber SH, Hendry AP (2008). A genetic assessment of polyandry and breeding-site fidelity in lemon sharks. Mol. Ecol.

[b15] Doyle JJ, Doyle JL (1987). A rapid DNA isolation procedure for small quantities of fresh leaf tissue. Phytochem. Bull.

[b16] Drummond AJ, Rambaut A (2007). BEAST: Bayesian evolutionary analysis by sampling trees. BMC Evol. Biol.

[b17] Drummond AJ, Ho SYW, Phillips MJ, Rambaut A (2006). Relaxed phylogenetics and dating with confidence. PLoS Biol.

[b18] Dudgeon CL, Broderick D, Ovenden JR (2009). IUCN classification zones concord with, but underestimate, the population genetic structure of the zebra shark *Stegostoma fasciatum* in the Indo-West Pacific. Mol. Ecol.

[b19] Edwards SV, Beerli P (2000). Perspective: gene divergence, population divergence, and the variance in coalescence time in phylogeographic studies. Evolution.

[b20] Edwards SV, Liu L, Pearl DK (2007). High-resolution species trees without concatenation. Proc. Natl Acad. Sci. USA.

[b21] Eschmeyer WN, Fricke H, Fong JD, Polack A (2010). Marine fish diversity: history of knowledge and discovery (Pisces). Zootaxa.

[b22] Felsenstein J (1981). Evolutionary trees from DNA sequences: a maximum likelihood approach. J. Mol. Evol.

[b23] Fitzpatrick JM, Carlon DB, Lippe C, Robertson DR (2011). The West Pacific diversity hotspot as a source or sink for new species? Population genetic insights from the Indo-Pacific parrotfish *Scarus rubroviolaceus*. Mol. Ecol.

[b24] Frey MA, Vermeij GJ (2008). Molecular phylogenies and historical biogeography of a circumtropical group of gastropods (Genus: *Nerita*): implications for regional diversity patterns in the marine tropics. Mol. Phylogenet. Evol.

[b25] Fu XY (1997). Statistical tests of neutrality of mutations against population growth, hitchhiking and background selection. Genetics.

[b26] Hall TA (1999). BioEdit: a user-friendly biological sequence alignment editor and analysis program for Windows 95/98/NT. Nucleic Acids Symp. Ser.

[b27] Harpending RC (1994). Signature of ancient population growth in a low-resolution mitochondrial DNA mismatch distribution. Hum. Biol.

[b28] Hillis DM, Huelsenbeck JP (1992). Signal, noise, and reliability in molecular phylogenetic analyses. J. Hered.

[b29] Johnson RH, Nelson DR (1978). Copulation and possible olfaction-mediated pair formation in two species of Carcharhinid sharks. Copeia.

[b30] Kajiura SM, Sebastian AP, Tricas TC (2000). Dermal bite wounds as indicators of reproductive seasonality and behaviour in the Atlantic stingray, *Dasyatis sabina*. Environ. Biol. Fishes.

[b31] Karl SA, Toonen RJ, Grant WS, Bowen BW (2012). Common misconceptions in molecular ecology: echoes of the modern synthesis. Mol. Ecol.

[b32] Kimura M (1980). A simple method for estimating evolutionary rates of base substitutions through comparative studies of nucleotid sequences. J. Mol. Evol.

[b33] Kimura M (1985). Back-arc rifting in the Okinawa Trough. Mar. Pet. Geol.

[b34] Larmuseau MHD, Raeymaekers JAM, Hellemans B, Volckaert JKJ, Van Houdt FAM (2010). Mito-nuclear discordance in the degree of population differentiation in a marine goby. Heredity.

[b35] Last PR, Stevens JD (2009). Sharks and rays of Australia.

[b36] Last PR, White WT, Last PR, White WT, Pogonoski JJ (2008). Resurrection of the genus *Neotrygon* Castelnau (Myliobatoidei: Dasyatidae) with the description of *Neotrygon picta* sp. nov., a new species from northern Australia. Descriptions of New Australian Chondrichthyans.

[b37] Last P, White WT, Puckridge M (2010). *Neotrygon ningalooensis* n. sp. (Myliobatoidei: Dasyatidae), a new maskray from Australia. Aqua, Int. J. Ichthyol.

[b38] Librado P, Rozas J (2009). DnaSP v5: a software for comprehensive analysis of DNA polymorphism data. Bioinformatics.

[b39] Liedloff A (1999). Mantel nonparametric test calculator. Version 2.0. School of natural resource sciences.

[b40] López AJ, Ryburn JA, Fedrigo O, Naylor GJP (2006). Phylogeny of sharks of the family Triakidae (Carchariniformes) and its implications for the evolution of carcharhiniform placental viviparity. Mol. Phylogenet. Evol.

[b41] Lourie SA, Vincent ACJ (2004). A marine fish follows Wallace's Line: the phylogeography of the three-spot seahorse (*Hippocampus trimaculatus*, Sygnathidae, Teleosti) in Southeast Asia. J. Biogeogr.

[b42] Lovejoy NR (1996). Systematics of myliobatoid elasmobranchs: with emphasis on the phylogeny and historical biogeography of neotropical freshwater stingrays (Potamotrygonidae: Rajiformes). Zool. J. Linn. Soc.

[b43] Manceau M, Domngues VS, Linnen CR, Rosenblum EB, Hoekstra HE (2010). Convergence in pigmentation at multiple levels: mutations, genes and function. Philos. Trans. R. Soc. B.

[b44] Manni F, Guérard E, Heyer E (2004). Geographic patterns of (genetic, morphologic, linguistic) variation: how barriers can be detected by “Monmonier's algorithm”. Hum. Biol.

[b45] Marmi J, Vila B, Oms O, Galobart À, Cappetta H (2010). Oldest records of stingray spines (Chondrichthyes, Myliobatiformes). J. Vertebr. Paleontol.

[b46] McMillan WO, Palumbi SR (1995). Concordant evolutionary patterns among Indo-West Pacific butterflyfishes. Proc. R. Soc. Lond. B Biol. Sci.

[b47] Mirams A, Treml E, Shields J, Liggins L, Riginos C (2011). Vicariance and dispersal across an intermittent barrier: population genetic structure of marine animals across the Torres Strait land bridge. Coral Reefs.

[b48] Moritz C (1994). Defining evolutionarily significant units for conservation. Trends Ecol. Evol.

[b49] Naylor GJP, Ryburn JA, Fedrigo O, López JA, Hamlett WC, Jamieson BGM (2005). Phylogenetic relationships among the major lineages of modern elasmobranches. Reproductive biology and phylogeny.

[b50] Palumbi SR, Hills DM, Moritz C, Mable BK (1996). Nucleic acids II: the polymerase chain reaction. Molecular systematics.

[b51] Pierce SJ, Pardo SA, Bennett MB (2009). Reproduction of the blue-spotted maskray *Neotrygon kuhlii* (Myliobatoidei: Dasyatidae) in south-east Queensland, Australia. J. Fish Biol.

[b52] Poore GCB, Andreakis N (2011). Morphological, molecular and biogeographic evidence support two new species in the *Uroptychus naso* complex (Crustacea: Decapoda: Chirostylidae). Mol. Phylogenet. Evol.

[b53] Portnoy DS, McDowell JR, Heist EJ, Musick JA, Graves JE (2010). World phylogeography and male-mediated gene flow in the sandbar shark, *Carcharhinus plumbeus*. Mol. Ecol.

[b54] Posada D, Crandall KA (1998). MODELTEST: testing the model of DNA substitution. Bioinformatics.

[b55] Puckridge M, Andreakis N, Appleyard SA, Ward RD (2013). Cryptic diversity in flathead fishes (Scorpaeniformes: Platycephalidae) across the Indo-West Pacific uncovered by DNA barcoding. Mol. Ecol. Res.

[b56] Rambaut A, Drummond AJ (2007). http://beast.bio.ed.ac.uk/Tracer.

[b57] Ramos-Onsins SE, Rozas J (2002). Statistical properties of new neutrality tests against population growth. Mol. Biol. Evol.

[b58] Read CI, Bellwood DR, van Herwerden L (2006). Ancient origins of Indo-Pacific coral reef fish biodiversity: a case study of the leopard wrasses (Labridae: *Macropharyngodon*. Mol. Phylogenet. Evol.

[b59] Renema W, Bellwood DR, Braga JC, Bromfield K, Hall R, Johnson KG (2008). Hopping hotspots: global shifts in marine biodiversity. Science.

[b60] Richards VP, Henning M, Witzell W, Shivji MS (2009). Species delineation and evolutionary history of the globally distributed spotted eagle ray (*Aetobatus narinari*. J. Hered.

[b61] Ronquist F, Huelsenbeck JP (2003). MrBayes 3: Bayesian phylogenetic inference under mixed models. Bioinformatics.

[b62] Rosenblum EB, Rompler H, Schoneberg T, Hoekstra HE (2010). Molecular and functional basis of phenotypic convergence in white lizards at White Sands. Proc. Natl Acad. Sci. USA.

[b63] Saint-Cast F, Condie SA (2006). Circulation modelling in Torres Strait.

[b64] Santini F, Winterbottom R (2002). Historical biogeography of Indo-western Pacific coral reef biota: is the Indonesian region a centre of origin?. J. Biogeogr.

[b65] Schluter SF, Marchalonis JJ (2003). Cloning of shark RAG2 and characterization of the RAG1/RAG2 gene locus. FASEB J.

[b66] Seehausen O, Mayhew PJ, van Alphen JJM (1999). Evolution of colour patterns in East African cichlid fish. J. Evol. Biol.

[b67] Shirai S, Stiassny MLJ, Parenti LR, Johnson GD (1996). Phylogentic interrelationships of neoselachians (Chondrichthyes: Euselachii). Interrelationships of fishes.

[b68] Sprintall J, Wijffels S, Molcard R, Jaya I (2009). Direct estimates of the Indonesian Throughflow entering the Indian Ocean: 2004–2006. J. Geophys. Res.

[b69] Swofford DL (2003). PAUP*. Phylogenetic analysis using parsimony (*and other methods). version 4.

[b70] Tamura K, Nei M (1993). Estimation of the number of nucleotide substitutions in the control region of mitochondrial DNA in humans and chimpanzees. Mol. Biol. Evol.

[b71] Theiss SM, Lisney TJ, Collin SP, Hart NS (2007). Colour vision and visual ecology of the blue-spotted maskray, *Dasyatis kuhlii* Müller & Henle, 1814. J. Comp. Physiol. A Neuroethol. Sens. Neural Behav. Physiol.

[b72] Tittensor DP, Mora C, Jetz W, Lotze HK, Ricard D, Berghe EV (2010). Global patterns and predictors of marine biodiversity across taxa. Nature.

[b73] Underwood CJ, Mitchell SF, Veltcamp KJ (1999). Shark and ray teeth from the Hauterivian (Lower Cretaceous) of north-east England. Palaeontology.

[b74] Voris HK (2000). Maps of Pleistocene sea levels in Southeast Asia: shorelines, river systems and time durations. J. Biogeogr.

[b75] Ward RD, Zemlak TS, Innes BH, Last P, Hebert PDN (2005). DNA barcoding Australia's fish species. Philos. Trans. R. Soc. B.

[b76] Ward RD, Holmes BH, White WT, Last PR (2008). DNA barcoding Australasian chondrichthyans: results and potential uses in conservation. Mar. Freshw. Res.

[b77] White WT, Dharmadi (2007). Species and size compositions and reproductive biology of rays (Chondrichthyes, Batoidea) caught in target and non-target fisheries in eastern Indonesia. J. Fish Biol.

[b78] White WT, Last PR, Stevens JD, Yearsley GK, Fahmi, Dharmadi (2006). Economically important sharks and rays of Indonesia.

[b79] Williams ST, Duda TF (2008). Did tectonic activity stimulate Oligo-Miocene speciation in the Indo-West Pacific?. Evolution.

[b80] Xia X, Xie Z (2001). DAMBE: software package for data analysis in molecular biology and evolution. J. Hered.

