# Supplementary material for: Phylogeography of the Indo-West Pacific maskrays (Dasyatidae, Neotrygon): a complex example of chondrichthyan radiation in the Cenozoic
Source: Ecol Evol. 2012 Dec 20;3(2):217–32. doi: 10.1002/ece3.448 (PMC3586632; doi:10.1002/ece3.448)

Supplementary Information - *PCR amplification*

PCR amplification reactions for the partial mitochondrial *cytochrome oxidase subunit I* (COI), the ribosomal 16S rDNA gene and a fragment of the nuclear single locus *recombination activating gene 1* (RAG1) spanning the highly variable 5`-end of the gene were performed in 20 µl reactions containing 2 µl 10x reaction buffer, 1.5-2.5 mM $MgCl_2$, 0.2 mM dNTPs, 10 mM of forward and reverse primers, 0.5 U of AmpliTaq and 10 – 20 ng template. Denaturation in 95 °C (10 mins) was followed by 35 cycles of a denaturation step of 94 °C (30 s), an annealing step of 50 °C, 54 °C or 57 °C for 16S, COI and RAG1 respectively for 30 s, an extension step of 72 °C (1 min) and a final extension of 72 °C (7 min). PCR products were quantified by agarose gel electrophoresis on a 1% TAE agarose gel using known standards. Amplicons were purified with AMPure XP (Agencourt) following manufacturer's recommendations and sequenced using the ABI Prism® BigDye® Terminator v3.1 cycle sequencing kit (Applied Biosystems).

Table S1 Specimens used in this study; accession numbers of sequences and collection information. Details of the COI haplotypes are also given for the specimens of *Neotrygon annotata*, *N. kuhlii* and *N. ningalooensis*.

Species	Sample #	COI	16s	RAG1	Collection location	Lat	Long	COI Haplotype
Neotrygon annotata	A11229	KC250628	KC249813	KC249767	Australia, QLD, Torres Strait	-10.5	142.4	Hap35
Neotrygon annotata	A11230	KC250622	KC249814	KC249768	Australia, QLD, Torres Strait	-10.5	142.4	Hap35
Neotrygon annotata	A11231	KC250623	KC249815	KC249769	Australia, QLD, Torres Strait	-10.5	142.4	Hap37
Neotrygon cf annotata	A2643	EU398731			Australia, QLD, Torres Strait	-10.5	142.4	Hap36
Neotrygon cf annotata	A2644	EU398730			Indonesia, West Java, Muara Angke	-6.1	106.8	Hap36
Neotrygon cf annotata	A2645	EU398729	KC249816		Indonesia, West Java, Muara Angke	-6.1	106.8	Hap36
Neotrygon cf annotata	A2646	EU398728	KC249817	KC249770	Indonesia, West Java, Muara Angke	-6.1	106.8	Hap36
Neotrygon cf annotata	A2647	EU398727	KC249818	KC249771	Indonesia, West Java, Muara Angke	-6.1	106.8	Hap36
Neotrygon kuhlii clade 1	A2584	EU398735	KC249819	KC249772	Taiwan, Penghu Islands	23.5	119.5	Hap1
Neotrygon kuhlii clade 1	A2585	EU398734	KC249820		Taiwan, Penghu Islands	23.5	119.5	Hap3
Neotrygon kuhlii clade 1	A2586	EU398733	KC249821		Taiwan, Penghu Islands	23.5	119.5	Hap1
Neotrygon kuhlii clade 1	A2587	KC250640	KC249822		Taiwan, Penghu Islands	23.5	119.5	Hap1
Neotrygon kuhlii clade 1	A6218	GU673431	KC249823	KC249773	Thailand, Gulf Coast	9.2	100.4	Hap1
Neotrygon kuhlii clade 1	A6219	GU673432	KC249824		Thailand, Gulf Coast	9.2	100.4	Hap1
Neotrygon kuhlii clade 1	A6276	GU673420	KC249825	KC249774	Vietnam, Con Son Island fish market	8.7	106.6	Hap2
Neotrygon kuhlii clade 1	A7817	HM902486	KC249826		Taiwan, Penghu Islands	23.5	119.5	Hap1
Neotrygon kuhlii clade 1	A7818		KC249827		Taiwan, Penghu Islands	23.5	119.5	-
Neotrygon kuhlii clade 1	A7819	HM902487	KC249828		Taiwan, Penghu Islands	23.5	119.5	Hap1
Neotrygon kuhlii clade 1	A7820		KC249829		Taiwan, Penghu Islands	23.5	119.5	-
Neotrygon kuhlii clade 1	BO409	KC249902	KC249830	KC249775	Malaysia, Sarawak	2.8	110.9	Hap2
Neotrygon kuhlii clade 2	A2575	EU398741	KC249831	KC249776	Indonesia, West Java, Muara Angke	-6.1	106.8	Hap6
Neotrygon kuhlii clade 2	A2576	EU398740	KC249832	KC249777	Indonesia, West Java, Muara Angke	-6.1	106.8	Hap4
Neotrygon kuhlii clade 2	A2577	EU398739	KC249833		Indonesia, West Java, Muara Angke	-6.1	106.8	Hap9
Neotrygon kuhlii clade 2	A2578	EU398738	KC249834		Indonesia, West Java, Muara Angke	-6.1	106.8	Hap7
Neotrygon kuhlii clade 2	A2579	EU398737	KC249835	KC249778	Indonesia, West Java, Muara Angke	-6.1	106.8	Hap8
Neotrygon kuhlii clade 2	A6221	GU673430	KC249836	KC249779	Thailand, Gulf Coast	9.2	100.4	Hap5
Neotrygon kuhlii clade 2	A7737	GU673709	KC249837		Indonesia, West Java, Muara Angke	-6.1	106.8	Hap9
Neotrygon kuhlii clade 2	BO424	KC249904	KC249838		Malaysia, Sarawak Tanjung Manis	2.1	111.1	Hap5

Neotrygon kuhlii clade 2	BO473	KC249905	KC249839	KC249780	Malaysia, Sarawak Mukah	3.0	112.0	Hap5
Neotrygon kuhlii clade 3	A6220	GU673429	KC249840	KC249781	Thailand, Gulf Coast	9.2	100.4	Hap10
Neotrygon kuhlii clade 3	BO423	KC249903	KC249841		Malaysia, Sarawak Tanjung Manis	2.9	112.1	Hap10
Neotrygon kuhlii clade 4	P-91858	AB485685			Japan, Okinawa, Ishigaki Is	24.4	124.0	Hap11
Neotrygon kuhlii clade 5	A208	DQ108184	KC249842	KC249782	Australia, QLD, Gulf of Carpentaria	-12.5	141.2	Hap14
Neotrygon kuhlii clade 5	A5649	KC250642	KC249843		Australia, QLD, Off Turtle Head Is	-10.5	142.4	Hap14
Neotrygon kuhlii clade 5	A5650	KC250645	KC249844		Australia, QLD, Torres Straits	-10.5	142.4	Hap14
Neotrygon kuhlii clade 5	A5960	KC250626	KC249845		Indonesia, Lombok, Tanjung Luar	-8.8	116.5	Hap16
Neotrygon kuhlii clade 5	A5961	KC250632	KC249846		Indonesia, Lombok, Tanjung Luar	-8.8	116.5	Hap16
Neotrygon kuhlii clade 5	A6198		KC249847	KC249783	Australia, WA, Shark Bay	-25.4	113.1	Hap12
Neotrygon kuhlii clade 5	A6199	GU673441	KC249848		Australia, WA, Shark Bay	-25.4	113.1	Hap15
Neotrygon kuhlii clade 5	A6849	KC250627	KC249849	KC249784	Australia, QLD, off Weipa	-12.5	141.2	Hap13
Neotrygon kuhlii clade 5	A6850	KC250635	KC249850		Australia, QLD, off Weipa	-12.5	141.2	Hap14
Neotrygon kuhlii clade 5	A7794	HM902468			Australia, QLD, off Weipa	-12.5	141.2	Hap14
Neotrygon kuhlii clade 6	A2571	EU398745	KC249851		Indonesia, Bali, Kedonganan	-8.8	115.2	Hap17
Neotrygon kuhlii clade 6	A2572	EU398744	KC249852		Indonesia, Bali, Kedonganan	-8.8	115.2	Hap17
Neotrygon kuhlii clade 6	A2573	EU398743			Indonesia, Bali, Kedonganan	-8.8	115.2	Hap17
Neotrygon kuhlii clade 6	A2574	EU398742	KC249853		Indonesia, Bali, Kedonganan	-8.8	115.2	Hap17
Neotrygon kuhlii clade 6	A2580	EF609342	KC249854	KC249785	Indonesia, Bali, Kedonganan	-8.8	115.2	Hap17
Neotrygon kuhlii clade 6	A2582	KC250629	KC249855		Indonesia, Bali, Kedonganan	-8.8	115.2	Hap17
Neotrygon kuhlii clade 6	A2583	EU398736	KC249856		Indonesia, Bali, Kedonganan	-8.8	115.2	Hap17
Neotrygon kuhlii clade 6	A5731	KC250630	KC249857	KC249786	Indonesia, Central Java, Sadang	-7.5	109.0	Hap17
Neotrygon kuhlii clade 6	A5737	KC250634	KC249858	KC249787	Indonesia, Central Java, Sadang	-7.5	109.0	Hap17
Neotrygon kuhlii clade 6	A5738	KC250639	KC249859		Indonesia, Central Java, Sadang	-7.5	109.0	Hap17
Neotrygon kuhlii clade 6	A5739	KC250637	KC249860	KC249788	Indonesia, Central Java, Sadang	-7.5	109.0	Hap17
Neotrygon kuhlii clade 6	A7812	HM902481	KC249861	KC249789	Indonesia, Bali, Kedonganan	-8.8	115.2	Hap17
Neotrygon kuhlii clade 7	A6222	GU673427	KC249862		Thailand, Andaman Coast	9.2	97.3	Hap18
Neotrygon kuhlii clade 7	A6223	GU673428	KC249863	KC249790	Thailand, Andaman Coast	9.2	97.3	Hap20
Neotrygon kuhlii clade 7	A6224	GU673425	KC249864		Thailand, Andaman Coast	9.2	97.3	Hap20
Neotrygon kuhlii clade 7	A6225	GU673426	KC249865	KC249791	Thailand, Andaman Coast	9.2	97.3	Hap19
Neotrygon kuhlii clade 7	A6226	GU673423	KC249866	KC249792	Thailand, Andaman Coast	9.2	97.3	Hap20
Neotrygon kuhlii clade 8	CHN 156	HM467799	HM467800		India, Kochi, Kerala	8.0	77.5	Hap21
Neotrygon kuhlii clade 8	80611	KC249906	KC249867	KC249793	Tanzania, Tanga	5.3	39.1	Hap22
Neotrygon kuhlii clade 9	A6217	GU673434	KC249868	KC249794	New Caledonia, Magenta Beach	-22.3	166.5	Hap24

Neotrygon kuhlii clade 9	A7791	HM902465	KC249869	KC249795	Australia, NSW, off Yamba	-29.3	153.3	Hap25
Neotrygon kuhlii clade 9	A7792	HM902466	KC249870		Australia, NSW	-29.3	153.3	Hap25
Neotrygon kuhlii clade 9	A7793	HM902467	KC249871		Australia, NSW	-29.3	153.3	Hap25
Neotrygon kuhlii clade 9	A7808	HM902478	KC249872	KC249796	Australia, QLD, Gladstone	-23.8	151.4	Hap28
Neotrygon kuhlii clade 9	A7809	HM902479	KC249873		Australia, QLD, Gladstone	-23.8	151.4	Hap25
Neotrygon kuhlii clade 9	A7810	HM902480	KC249874		Australia, QLD, Gladstone	-23.8	151.4	Hap26
Neotrygon kuhlii clade 9	A7811		KC249875		Australia, QLD, Gladstone	-23.8	151.4	
Neotrygon kuhlii clade 9	A7813	HM902482	KC249876	KC249797	Australia, QLD, Moreton Bay	-27.2	153. 3	Hap25
Neotrygon kuhlii clade 9	A7814	HM902483	KC249877		Australia, QLD, Moreton Bay	-27.2	153. 3	Hap27
Neotrygon kuhlii clade 9	A7815	HM902484	KC249878		Australia, QLD, Moreton Bay	-27.2	153. 3	Hap25
Neotrygon kuhlii clade 9	A7816	HM902485	KC249879		Australia, QLD, Moreton Bay	-27.2	153. 3	Hap25
Neotrygon kuhlii clade 9	UGA008	KC250643			Australia, QLD, Lizard Island	-14.7	145.5	Hap23
Neotrygon leylandi	A2917	EU398748	KC249880	KC249798	Australia, WA, Exmouth	-22.0	114.0	
Neotrygon leylandi	A2935	EU398747	KC249881	KC249799	Australia, WA, Shark Bay	-25.4	113.1	
Neotrygon leylandi	A6235	KC250638	KC249882		Australia, WA, Coral Bay	-23.1	113.4	
Neotrygon picta	A5653	KC250624	KC249883	KC249800	Australia, QLD, Torres Strait	-10.5	142.4	
Neotrygon picta	A5659	KC250641	KC249884	KC249801	Australia, QLD, Torres Strait	-10.5	142.4	
Neotrygon picta	A5661	KC250625	KC249885		Australia, QLD, Torres Strait	-10.5	142.4	
Neotrygon ningalooensis	A6233	HQ955938	KC249886	KC249802	Australia, WA, Coral Bay	-23.1	113.4	Hap34
Neotrygon ningalooensis	A6234	KC250644	KC249887	KC249803	Australia, WA, Coral Bay	-23.1	113.4	Hap34
Neotrygon ningalooensis	A6205	GU673440	KC249888	KC249804	Australia, WA, Shark Bay	-25.4	113.1	Hap32
Neotrygon ningalooensis	A6215	GU673436	KC249889		Australia, WA, Shark Bay	-25.4	113.1	Hap30
Neotrygon ningalooensis	A6216	GU673433	KC249890		Australia, WA, Shark Bay	-25.4	113.1	Hap29
Neotrygon ningalooensis	A6204	GU673439			Australia, WA, Shark Bay	-25.4	113.1	Hap32
Neotrygon ningalooensis	A6206	GU673437			Australia, WA, Shark Bay	-25.4	113.1	Hap31
Neotrygon ningalooensis	A6207	GU673438			Australia, WA, Shark Bay	-25.4	113.1	Hap31
Neotrygon ningalooensis	A6212	GU673435			Australia, WA, Shark Bay	-25.4	113.1	Hap33
Outgroups								
Dasyatis brevicaudata		EU398726	EU848428					
Dasyatis parvonigra	A3755	EU398732	KC249891	KC249805				
Dasyatis ushiei	BW-2176	EU398753	KC249892	KC249806				
Dasyatis zugei	A3765	EU398759						
Dasyatis zugei	A3766	EU398758	KC249893					
Himantura astra	A225	DQ108171	KC249894					

Himantura walga		EU398876	KC249895	KC249807				
Pastinachus atrus	A2161	EU398970	KC249896	KC249808				
Pteroplatytrygon violacea	HM239671	HM239671	HM239658					
Taeniura lymma	UG0012	KC250631	KC249897	KC249809				
Taeniura lymma	UG0713	KC250633	KC249898	KC249810				
Taeniurops meyeni	A6227	GU6737424	KC249899	KC249811				
Urogymnus asperrimus	A4563	KC250636	KC249900	KC249812				
Rhynchobatus australiae	A2380	EU399007	KC249901					
Rhynchobatus australiae	A2925	EU399008						
Carcharhinus leuca	1470116			U62645				

*All Axxxx numbers refer to corresponding Barcode of Life Database specimen numbers BW-Axxxx

Table S2 Kimura 2-Parameter (K2P) corrected Neighbour Joining distance matrix with minimum and maximum values (in brackets) for the *Neotrygon kuhlii* mitochondrial COI clades 1 to 9.

Clade	1	2	3	4	5	6	7	8	9
1	**0.11 (0.00-0.46)**								
2	2.07 (1.71-2.4)	**0.58 (0-0.93)**							
3	2.03 (1.89-2.19)	1.33 (1.09-1.56)	**0 (0-0)**						
4	3.11 (2.99-3.16)	2.54 (2.31-2.83)	2.48 (2.48-2.48)	**N/A**					
5	3.47 (3.16-3.72)	3.16 (2.70-3.57)	3.10 (3.00-3.23)	3.47 (3.34-3.86)	**0.53 (0-1.4)**				
6	3.29 (3.16-3.49)	2.61 (2.36-2.93)	2.53 (2.52-2.59)	3.17 (3.17-3.27)	2.68 (2.36-2.96)	**0 (0-0)**			
7	3.42 (3.22-3.56)	2.35 (2.04-2.68)	2.33 (2.24-2.36)	3.31 (3.23-3.34)	2.84 (2.40-3.07)	1.69 (1.59-1.77)	**0.16 (0.00-0.31)**		
8	3.29 (2.67-3.89)	2.56 (1.88-3.34)	2.36 (2.04-2.68)	3.17 (2.65-3.69)	2.53 (1.88-4.81)	1.41 (0.77-2.1)	1.58 (1.11-2.08)	**1.56 (1.56-1.56)**	
9	4.62 (4.18-4.90)	4.05 (3.65-4.48)	4.00 (3.82-4.15)	3.57 (3.36-3.84)	4.53 (3.97-5.15)	3.97 (3.76-4.23)	4.07 (3.72-4.32)	3.8 (0.02-4.32)	**0.3 (0.00-0.93)**

Table S3 K2P-corrected neighbour-joining distance matrix of *Neotrygon* species calculated from the COI alignment. Genetic distance is along the lower diagonal and standard error along the upper diagonal.

N. annotata		0.20	1.54	1.58	1.49	1.55	1.49	1.51	1.50	1.56	1.61	1.65	1.64	1.61	1.56
N. cf annotata	0.46		1.57	1.58	1.51	1.56	1.50	1.53	1.51	1.55	1.62	1.64	1.64	1.61	1.57
N. ningalooensis	12.72	13.05		0.80	1.38	1.38	1.47	1.37	1.38	1.50	1.43	1.38	1.38	1.36	1.38
N. cf ningalooensis	14.14	13.94	4.75		1.47	1.52	1.50	1.34	1.37	1.52	1.49	1.38	1.35	1.37	1.39
N. leylandi	12.72	12.92	11.57	12.24		0.63	1.31	1.33	1.29	1.37	1.31	1.32	1.42	1.29	1.38
N. picta	13.13	13.33	11.49	12.16	2.73		1.30	1.28	1.24	1.33	1.29	1.32	1.32	1.31	1.31
N. kuhlii clade 1	12.77	12.83	12.25	13.52	10.08	10.31		0.51	0.55	0.73	0.72	0.75	0.74	0.67	0.81
N. kuhlii clade 2	12.64	12.58	10.84	11.69	10.39	10.25	2.07		0.42	0.60	0.66	0.62	0.55	0.55	0.72
N. kuhlii clade 3	12.39	12.32	10.98	11.84	10.08	9.94	2.03	1.33		0.61	0.68	0.64	0.60	0.57	0.76
N. kuhlii clade 4	12.97	12.63	12.22	12.94	10.65	10.64	3.11	2.54	2.48		0.76	0.75	0.74	0.69	0.69
N. kuhlii clade 5	13.48	13.36	11.96	13.07	10.06	10.30	3.47	3.16	3.10	3.47		0.66	0.65	0.56	0.77
N. kuhlii clade 6	13.83	13.50	11.36	11.84	10.45	10.69	3.29	2.61	2.53	3.17	2.68		0.48	0.40	0.73
N. kuhlii clade 7	13.95	13.61	11.21	11.69	10.84	10.57	3.42	2.35	2.33	3.31	2.84	1.69		0.40	0.74
N. kuhlii clade 8	13.53	13.20	11.17	11.65	10.26	10.69	3.29	2.56	2.36	3.17	2.53	1.41	1.58		0.69
N. kuhlii clade 9	12.93	12.86	10.81	11.67	10.20	9.82	4.62	4.05	4.00	3.57	4.53	3.97	4.07	3.81	

Table S4
Mean divergence times and their 95% HPD confidence intervals based on fossil calibrations and published COI mutation rates (0.58% / My and 0.9% / My) calculated in the spotted eagle ray (*A. narinari*) from the isthmus of panama closure for nodes 1 to 10.

	Fossil		Mutation rate			
			0.58% / My		0.9% / My	
Node	Age	(95% CI)	Age	(95% CI)	Age	(95% CI)
1	69.9	(51.5-93.2)	7.9	(12.6-24.3)	11.6	(8.0-16.0)
2	54.2	(38.6-71.8)	3.9	(9.8-19.2)	9.0	(6.1-12.5)
3	46.7	(32.8-62.3)	1.2	(7.7-15.6)	7.3	(5.0-10.1)
4	35.4	(23.5-49.3)	8.9	(5.9-12.6)	5.8	(4.0-8.2)
5	15.7	(9.45-23.7)	2.9	(1.9-4.4)	1.9	(1.2-3.0)
6	10.2	(4.1-12.3)	2.0	(1.2-3.2)	1.4	(0.8-2.1)
7	10.7	(6.2-16.7)	2.3	(1.3-3.4)	1.5	(0.9-2.3)
8	11.4	(5.4-18.3)	2.9	(1.3-5.1)	1.9	(0.9-3.3)
9	10.7	(4.5-18.3)	3.0	(1.3-5.8)	2.0	(0.8-3.9)
10	4.7	(1.9-8.3)	0.8	(0.2-1.8)	0.5	(0.2-1.2)

Mutation rates from the present study

COI = 0.229% per My
16s = 0.07835% per My
RAG1 = 0.02231% per My

Figure S1

Substitution frequencies of transitions (cross) and transversions (triangle) for: a) the 1st, b) 2nd and c) 3rd codon positions of the partial COI gene. Observed frequencies are calculated with the K2P genetic distance.

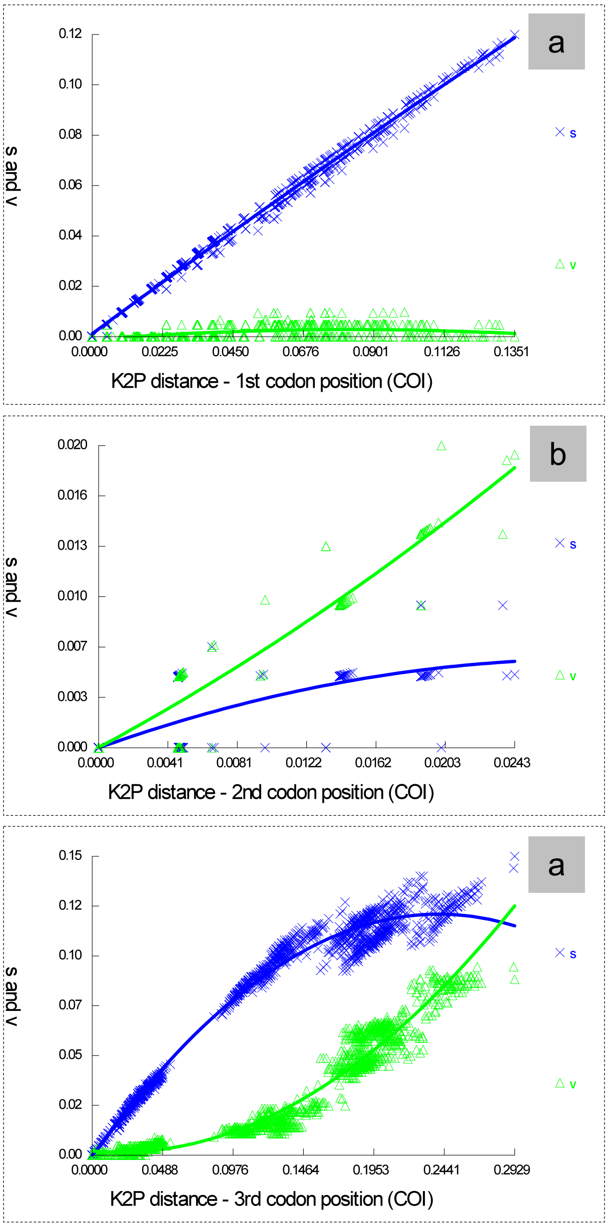

Figure S2
Substitution frequencies of transitions (cross) and transversions (triangle) for: a) the 1st, b) 2nd and c) 3rd codon positions of the partial 16s gene. Observed frequencies are calculated with the K2P genetic distance.

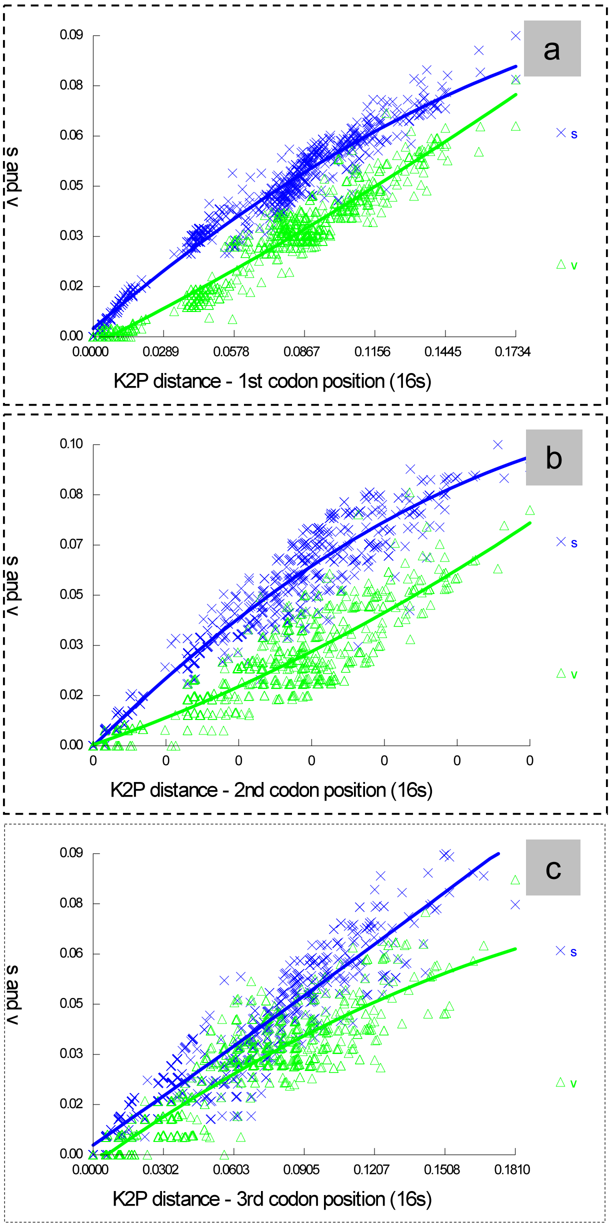

Figure S3
Substitution frequencies of transitions (cross) and transversions (triangle) for: a) the 1st, b) 2nd and c) 3rd codon positions of the partial RAG1 gene. Observed frequencies are calculated with the K2P genetic distance.

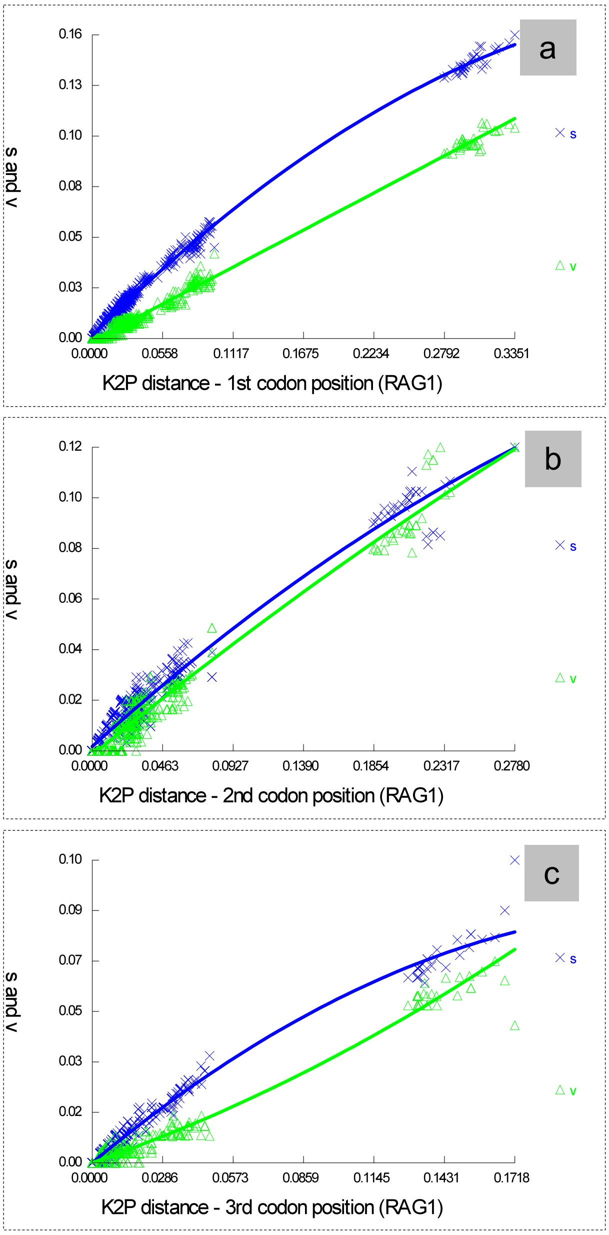

Figure S4

Mismatch distributions for pairwise comparisons of *Neotrygon kuhlii* COI mitochondrial clades. Monomorphic clades 3, 4 and 6 were excluded from the calculation. The frequencies of expected and observed differences are given as a solid and dashed line, respectively.

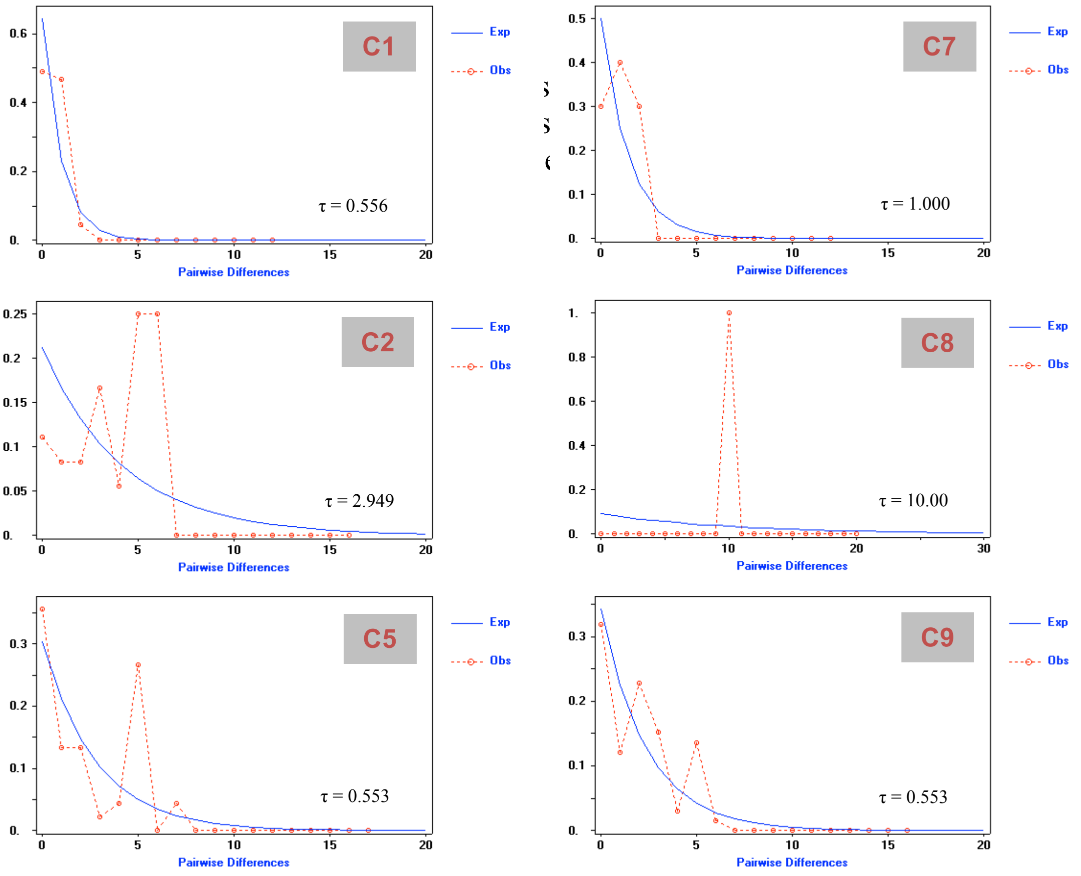

Supplement: Supplementary file 1 [file ece30003-0217-SD1.pdf]
